# Comparative econometric cost analysis of inpatient versus outpatient laser treatment for twin-twin transfusion syndrome

**DOI:** 10.61622/rbgo/2026rbgo61

**Published:** 2026-08-20

**Authors:** Cleisson Fábio Andrioli Peralta, Klenio Barbosa, Raquel Pereira Pontes, Karina Jorge Rodrigues da Costa, Samara Pinheiro do Carmo Gomes, Elton Sousa Santos, Letícia Galvão Barbante

**Affiliations:** 1 Hospital do Coração São Paulo SP Brazil Hospital do Coração, São Paulo, SP, Brazil.; 2 Gestar Centro de Medicina e Cirurgia Fetal São Paulo SP Brazil Gestar Centro de Medicina e Cirurgia Fetal, São Paulo, SP, Brazil.; 3 Centro de Treinamento em Ultrassonografia São Paulo SP Brazil Centro de Treinamento em Ultrassonografia, São Paulo, SP, Brazil.; 4 Université Cote d’Azur SKEMA Business School Sophia Antipolis France SKEMA Business School, Université Cote d’Azur, Sophia Antipolis, France.; 5 Universidade do Vale do Rio dos Sinos São Leopoldo RS Brazil Universidade do Vale do Rio dos Sinos, São Leopoldo, RS, Brazil.

**Keywords:** Twins, monozygotic, Fetofetal transfusion, Inpatients, Outpatients, Patient discharge, Pregnant women, Pregnancy, twin, Laser therapy, Costs and cost analysis, Cost-benefit analysis

## Abstract

**Objective:**

To compare the costs of inpatient versus outpatient laser ablation of placental vessels treatment of severe twin-twin transfusion syndrome. To evaluate if outpatient is non-inferior to inpatient treatment with regards to feasibility and safety.

**Methods:**

Case-control study of 71 pregnant women who underwent laser ablation of placental vessels for treatment of severe twin-twin transfusion syndrome between 2019 and 2022. To compare the costs of outpatient versus inpatient treatments we used conditional estimation of the average costs of each procedure and the differences between them with paired data estimators: multivariate distance matching and propensity score matching. Comparisons of surgical and clinical variables were performed using the Chi-square or Fisher's exact tests and Student's t-test.

**Results:**

Laser ablation of placental vessels was performed in 71 patients with severe twin-twin transfusion syndrome. Sixteen (16/71 – 23%) patients were operated on in the outpatient clinic and 55 (55/71 – 77%) were operated on as inpatients. The cost of inpatient intervention was 28% and 29% higher than the cost of the outpatient treatment, according to the multivariate distance matching and propensity score matching estimates, respectively. Apart from ex-recipient intrauterine death (greater in the outpatient group), there were no significant differences between the two groups regarding laser ablation of placental vessels, post laser treatment, perinatal variables and variables from delivery until hospital discharge.

**Conclusion:**

Laser ablation of placental vessels to treat severe twin-twin transfusion syndrome in an outpatient setting is significantly cheaper than in inpatients, without any compromise in feasibility and safety, as most fetal, neonatal and maternal results were similar.

## Introduction

Twin-twin transfusion syndrome (TTTS) is a severe condition that affects about 10-15% of monochorionic-diamniotic (MCDA) twin pregnancies and results from unbalanced blood flow between the fetuses, mainly through arterio-venous placental anastomosis.^(1,2)^ It is life-threatening for the twins, and short- and long-term prognosis for the mother and the fetuses are significantly compromised by inappropriate follow-up and/or management.

The treatment of choice for severe TTTS (stages II-IV in the classification of Quintero et al.^(1)^) is the laser ablation of placental vessels (LAPV).^(1-4)^ This intervention can be performed under local anesthesia on an outpatient clinic, or under locoregional block, which requires hospitalization and the presence of an anesthetist during the procedure. The latter approach probably increases the costs of treatment in both public and private health systems, but not necessarily improves the gestational results.

The main objective of this study was to compare the costs of outpatient versus inpatient treatments of severe TTTS using LAPV. Moreover, it was evaluated if outpatient is non-inferior to inpatient laser treatment with regards to feasibility and safety.

## Methods

This was a case-control study about pregnant women who underwent LAPV for the treatment of severe TTTS between 2019 and 2022 at the Heart Hospital in São Paulo, Brazil.

The inclusion criteria for LAPV were MCDA twin pregnancies with severe TTTS (stages II-IV in the classification by Quintero et al.^(1)^), without fetal major anatomical defects (twin reverse arterial perfusion sequence or any other major fetal abnormality) or chromosomal abnormalities, and a cervical length greater than 15 mm (the 5^th^ percentile according to To et al.^(5)^) before the procedure.

All interventions were performed by the same surgeon (CFAP) according to previously published technique.^(3,4)^ Briefly, the procedure was conducted as follows: the placental chorionic plate vessels through the amniotic cavity of the recipient fetus were initially mapped endoscopically; the vascular equator was identified; a line of ablation of the chorionic plate was created from one edge of the placenta to the other, including the arterio-venous anastomoses and vessels with unknown courses. At the end of LAPV, the excess amniotic fluid was drained through the fetoscopy sheath to ensure that the deepest amniotic fluid pocket measured less than 8 cm. From 2019 to 2021 all patients were operated on under locoregional spinal anesthesia and in 2022 all patients were operated on under local anesthesia on an outpatient basis, i.e. without hospitalization.

The following clinical and surgical variables were evaluated: LAPV variables: gestational age (GA), the stage of TTTS, cervical length, type of maternal anesthesia (locoregional or local), the need to change the anesthetic technique, total surgical time, and the occurrence of maternal and/or fetal complications; Post LAPV variables: the rates of intrauterine death of the ex-donor and ex-recipient twins until delivery and the rates of premature preterm rupture of membranes (PPTRM); Perinatal variables: GA at birth, birthweight (BW), Apgar scores, the occurrence of maternal and neonatal complications; Variables from delivery until hospital discharge: among liveborn twins, the rates of hospital discharge of the ex-donors and ex-recipients.

The following variables were collected to evaluate the costs of the LAPV procedures: total costs for each patient, which were disaggregated into 13 expense categories: daily allowances, imaging exams, laboratory exams, physiotherapy and rehabilitation, cleaning and laundry materials, medical materials, medicines, special materials, room fee, additional fees, clinical nutrition, office supplies and medical fees. The doctors involved in the project did not charge fees for the procedures. As there is no Brazilian Medical Association (BMA) code for the LAPV, an approximate estimation of this cost was used, considering the medical costs for a procedure of similar complexity, which has a code in the BMA table.

### Statistical analysis

For clinical and surgical variables, averages and standard deviations (SD) were used to describe continuous variables, and absolute and relative frequencies were calculated to describe categorical data. Comparisons of categorical variables were performed using the Chi-square or Fisher's exact tests. Continuous variables were compared using Student's t-test.

### Cost analysis

The differences in costs between the two groups (inpatient x outpatient) were estimated using matching estimation techniques. Two pairwise estimation methods were used, the multivariate distance matching (MDM) and the propensity score matching (PSM). In both, the patient's age and the type of health plan (public system x private medical insurance) were used in the pairing.

A p-value of less than 5% was considered statistically significant. The Statistical Package for the Social Sciences (SPSS, Chicago, IL, USA), version 29.0, was used for the analyses.

The study was approved by the research ethics committee of the Heart Hospital (approval number: 5.036.183) and is part of a larger project that aims to evaluate maternal and children's outcomes after intrauterine surgeries (CAAE: 52159721.3.0000.0060).

## Results

Laser ablation of placental vessels was performed in 71 patients with severe TTTS who met the entry criteria. Sixteen (16/71 – 23%) patients were operated on in the outpatient clinic under local anesthesia and 55 (55/71 – 77%) were operated on as inpatients under locoregional anesthesia. There were no significant differences between the two groups regarding maternal age [Average (SD) - Inpatient: 29.7 years (6.0) / outpatient: 29.8 years (5.2) / p: 0.941], parity [Nulliparity/total (%) - Inpatient: 31/55 (56)/ outpatient: 11/16 (69) / p: 0.375], and a composite of other maternal clinical conditions (diabetes, chronic hypertension, hypo/hyperthyroidism, autoimmune disease, other) [Maternal clinical condition/total (%) - Inpatient: 7/55 (13)/ outpatient: 3/16 (19) / p: 0.542]. There were no maternal or fetal complications during the LAPV. None of the procedures performed in the outpatient clinic had to be converted to locoregional anesthesia. Table 1 demonstrates that, apart from ex-recipient IUD, which was greater in the outpatient group, there were no other significant differences between the two groups regarding LAPV, post LAPV, perinatal variables and variables from delivery until hospital discharge.

**Table 1:** Comparison of inpatient x outpatient treatments regarding LAPV, post LAPV, perinatal variables and variables from delivery until hospital discharge

Variables	Inpatient n= 55	Outpatient n= 16	p-value
LAPV			
Gestational age, weeks: Average (SD)	21.16 (2,44)	21.80 (2.42)	0.673
Quintero stage: n/total (%)	II: 23/55 (42)	II: 5/16 (31)	0.366
	III: 28/55 (51)	III: 8/16 (50)	
	IV: 4/55 (7)	IV: 3/16 (19)	
Cervical length, mm: Average (SD)	32.7 (8.5)	29.0 (10.20)	0.243
Surgical time, min.: Average (SD)	45.8 (14.3)	51.1 (14.1)	0.826
Post-LAPV			
Ex-donor IUD: n/total (%)	12/55 (22)	5/16 (31)	0.221
Ex-recipient IUD: n/total (%)	6/55 (11)	6/16 (38)	0.012
PPTRM: n/total (%)	14/50 (28)	3/11 (27)	0.638
Perinatal			
Gestational age at birth, weeks: n; Average (SD)	50; 31.5 (3.6)	11; 32.1 (3.5)	0.763
Birthweight, g.: Average (SD)			
Ex-donor	1424 (606)	1540 (599)	0.582
Ex-recipient	1666 (663)	1634 (340)	0.824
Apgar scores: Average (SD)			
Ex-donor			
1^o^ min.	7.0 (1.8)	6.2 (1.4)	0.180
5^o^ min.	8.7 (1.0)	8.7 (1.0)	0.976
Ex-recipient			
1^o^ min.	7.0 (1.7)	6.1 (1.9)	0.164
5^o^ min.	8.4 (1.1)	8.1 (1.1)	0.444
Delivery to hospital discharge			
Hospital discharge: n/total* (%)			
Ex-donor	37/46 (80)	9/10 (90)	0.424
Ex-recipient	38/47 (81)	9/10 (90)	0.435

LAPV: Laser ablation of placental vessels; SD: Standard deviation; IUD: Intrauterine death; PPTRM: Premature preterm rupture of membranes

* liveborn twins

Table 2 shows a comparison of total costs and costs by category. Table 3 shows that treatment costs were 28% and 29% more expensive (Brazilian reais) for inpatients, according to the MDM and PSM estimates, respectively.

**Table 2:** Comparison between inpatient x outpatient LAPV treatments of severe TTTS regarding total costs and costs by category.

Variables*	Inpatient n=55	Outpatient n=16	Difference	p-value
Total:	7724.84 (6863.73 – 8585.95)	5696.01 (4436.27 – 6955.76)	2028.82 (314.23 – 374.42)	<0.001
Daily allowances	3427.40 (2906.94 – 3947.86)	2612.84 (1514.27 – 3711.40)	814.56 (-293.63 – 1922.75)	0.15
Imaging exams	6.04 (-1.18 – 13.26)	0.00 (0.00 – 0.00)	6.04 (-7.36 – 19.43)	0.37
Laboratory exams	37.28 (6.79 – 67.76)	5.76 (2.71 – 8.81)	31.51 (-25.03 – 88.06)	0.27
Physiotherapy and rehabilitation	7.42 (-4.10 – 18.95)	0.00 (0.00 – 0.00)	7.42 (-13.94 – 28.79)	0.49
Cleaning and laundry supplies	1.72 (1.17 – 2.26)	2.69 (1.78 – 3.59)	-0.97 (-2.07 – 0.12)	0.08
Medical supplies	912.37 (826.53 – 998.20)	691.91 (532.19 – 851.62)	220.46 (42.68 – 398.23)	0.02
Medications	294.67 (261.14 – 328.20)	217.18 (170.05 – 264.29)	77.50 (11.08 – 143.91)	0.02
Special materials	36.53 (-0.26 – 1.47)	0.00 (0.00 – 0.00)	36.53 (-1.01 – 2.,22)	0.48
Room fee	1965.13 (1387.98 – 2542.28)	1402.79 (1013.88 – 1791.70)	562.34 (-525.11 – 1649.79)	0.31
Additional fees	3.39 (-1.23 – 8.02)	0.00 (0.00 – 0.00)	3.39 (-5.19 – 11.97)	0.43
Clinical nutrition	0.14 (-0.14 – 0.42)	1.08 (0.02 – 0.19)	-0.94 (-1.43 – -0.85)	0.03
Office supplies	0.11 (0,02 – 0.19)	1.25 (0.75 – 1.75)	-1.15 (-1.43 – -0.85)	<0.001
Medical fees	1032.62 (1027.37 – 1037.88)	760.50 (760.50 – 760.50)	272.12 (262.38 – 281.87)	<0.001

* For all variables, results are presented in Brazilian Reais: Average (95% CI); LAPV: Laser ablation of placental vessels; TTTS: Twin-twin transfusion syndrome.

**Table 3:** Comparison between inpatient x outpatient LAPV treatments of severe TTTS regarding total costs, according to the MDM and PSM estimates

	Total cost		Ln Total cost	
	MDM, Average (95% CI)	PSM, Average (95% CI)	MDM, Average (95% CI)	PSM, Average (95% CI)
Outpatient – inpatient costs	-2057.23 (-3494.89 – -619.56)	-2069.46 (-3803.89 – -335.03)	-0.28 (-0.46 – 0.10)	-0.28 (-0.52 – -0.08)
Standard error	720.83	869.63	0.09	0.10
T statistics	-2.85	-2.38	-3.15	-2.79
p-value	0.006	0.020	0.002	0.007
Average estimated costs* Inpatient Outpatient	7724.84 5667.61	7724.84 5655.38		

* Results are presented in Brazilian Reais: Average (95% CI); LAPV: Laser ablation of placental vessels; TTTS: Twin-twin transfusion syndrome; MDM: Multivariate Distance Matching (Mahalanobis distance metrics); PSM: Propensity Score Matching

## Discussion

This study demonstrated that LAPV treatment of severe TTTS is significantly cheaper in an outpatient setting with local anesthesia than in inpatients using spinal anesthesia. It was also demonstrated that outpatient treatment was not inferior to inpatient treatment with regards to feasibility and safety, as most fetal, neonatal and maternal results were not statistically different.

Endoscopic LAPV is the treatment of choice for severe TTTS (stages II to IV in the classification by Quintero et al.^(1)^).^(1-4)^ About treatment settings, the options to treat according to inpatient or outpatient protocols, using local or locoregional anesthesia, is a matter of preference and varies considerably among centers. In the present study, despite understandable, treatment costs were significantly lower for outpatients, without any significant impact in surgical and/or clinical results. We did not run a formal cost-effectiveness analysis, however, the observation that surgical and clinical outcomes were similar in both groups allows an indirect inference that the most cost-effective approach would be in an outpatient basis. When it comes to cost analysis and cost-benefit, the available literature is restricted to comparisons of different modalities of treatment for severe TTTS, basically among LAPV, serial amniodrainage and septostomy.^(6,7)^ A cost-effectiveness analysis of treatment strategies demonstrated that, for stage I TTTS, expected management had the lowest cost and the highest rate of at least one survival.^(6)^ This study demonstrated that LAPV was the most cost-effective in the treatment of stages II – IV TTTS.^(6)^ This had already been highlighted in a study by Odibo et al.^(7)^

Considering the surgical and clinical results in the present study, apart from a higher IUD of the ex-recipient twin in the outpatient group, there were no significant differences between inpatient and outpatient treatments. In addition, some comparisons of our results with available literature deserve comments. Our fetal results were like those previously published, but our neonatal outcomes were worse in some variables. Pooled data from a recent metanalysis demonstrated that fetal loss ranged from 15 to 19%, neonatal death ranged from 2 to 5%, survival of at least one twin ranged from 85 to 92% and double survival ranged from 62 to 73%.^(8-15)^ Our results were respectively 20%, 14%, 76% and 53%. Differences in the survival of at least one twin and double survival were influenced by the higher rates of neonatal death, which probably reflects an inferior quality of neonatal assistance in our country. Patients operated on in our hospital return to their referring centers for follow-up and delivery and our country is marked by a great heterogeneity of medical assistance's quality. About pregnancy complications, the same above mentioned metanalysis demonstrated rates of PPTROM ranging from 17 to 22%, maternal bleeding ranging from 6.5 to 9.5%, septostomy or chorioamniotic separation ranging from 13 to 14% and chorioamnionitis ranging from to 1.2 to 2.7%.^(8)^ In our study, the same complications occurred respectively in 24%, 0%, 11% and 0% of patients.

The main limitations in the present study were the small number of cases, the fact that inpatient and outpatient groups were not contemporaneous and the lack of uniformity in the perinatal and neonatal managements. One positive aspect was that all LAPV were performed in the same hospital and by the same operator, using the same LAPV technique.

## Conclusion

LAPV to treat severe TTTS in an outpatient setting is significantly cheaper than in inpatients, without any compromise in feasibility and safety, as most fetal, neonatal and maternal results were similar.

## Data Availability

The research data are described in the article presented.
